# Supplementation with protected kapok seed oil and choline chloride to improve the performance and lipid status of thin-tailed sheep

**DOI:** 10.14202/vetworld.2023.1520-1526

**Published:** 2023-07-24

**Authors:** Widiyanto Widiyanto, Mulyono Mulyono, Bambang Waluyo Hadi Eko Prasetiyono

**Affiliations:** Department of Animal Science, Faculty of Animal and Agricultural Sciences, Diponegoro University, Tembalang Undip Campus, Semarang, Indonesia

**Keywords:** cholesterol, linoleic acid, male thin-tailed sheep, meat, performance

## Abstract

**Background and Aim:**

Healthy meat production is an important aspect of increasing sheep productivity. This study aimed to examine the influence of protected kapok seed oil (KSO) in combination with choline chloride (CC) on the feed utilization, lipid status, and performance of thin-tailed sheep.

**Materials and Methods:**

Thirty male thin-tailed sheep (approximately 6 months old, with an average body weight of 12.59 ± 1.48 kg) were divided into six treatment groups (five heads/treatment). Factor 1 consisted of two treatments: K_1_ (KSO supplementation at 10% supplementation and 75% protection level) and K_0_ (without KSO supplementation). Factor 2 consisted of three levels of CC: (C_0_: 0%; C_1_: 1.5% and C_2_: 3% feed dry matter (DM) basis supplementation levels). The variables measured were the DM consumption, DM digestibility, organic matter digestibility, nitrogen retention, daily body weight gain (DBWG), and blood and meat lipid status. The data were analyzed using analysis of variance in a completely randomized design in a factorial pattern of 2 × 3 × 5.

**Results:**

Choline chloride supplementation (up to 3%) increased DM consumption in the K_0_C_2_ group. The CC and protected KSO (K_1_C_2_) supplementation combination resulted in the highest DM consumption level (p < 0.05). The protected KSO supplementation increased DBWG (the DBWG in the K_1_C_0_ group was higher than that in the K_0_C_0_ group, and the highest DBWG was found in the K_1_C_2_ group) (p < 0.05). Protected KSO and CC supplementation decreased cholesterol levels and increased the relative proportion of linoleic acid in meat (p < 0.05).

**Conclusion:**

Combined supplementation with protected KSO and CC improved the feed utilization and performance of male thin-tailed sheep. There were increases in DBWG, decreases in intramuscular fat and cholesterol levels, and increases in meat linoleic acid levels.

## Introduction

One of the constraints in efforts to increase the productivity of small ruminants is the low consumer preferences for ruminant meat due to cholesterol phobia in the community. This problem needs to be overcome through education and technology to improve the quality of small ruminant meat while increasing its productivity [1].

Supplementation of protected polyunsaturated fatty acid sources in concentrate can increase meat productivity with a healthy lipid status (low cholesterol and rich in omega-6 fatty acids) [1]. The widespread application of this technology in the field can increase consumer preferences for small ruminant meat and encourage increased productivity so that it has a significant role in achieving the target of meat self-sufficiency.

As an illustration, the research of Widiyanto *et al*. [2] showed that supplementation with 10% kapok seed oil (KSO) with a protection level of 75% in the ration of thin-tailed sheep resulted in a decrease in cholesterol levels of thin-tailed sheep meat from 89.21 mg to 62.46 mg/100 g and an increase in the relative proportion of omega-6 fatty acids from 3.68% to 15.52%. The weight gain of sheep increased from an average of 62 g/day–99 g/day. There was a weaker increase in the meat fat content from 3.04% to 5.93%. Based on the results of the above analysis, it is necessary to conduct research by combining the treatment of protected KSO supplementation with choline chloride (CC).

Choline is a source of methyl groups for the synthesis of phosphatidyl choline, which is an important component of cell membranes [3]. These compounds play a role in maintaining membrane fluidity and integrity, thereby increasing the entry of nutrients into cells as well as stimulating intracellular metabolism and increasing the biosynthesis of livestock products, which is reflected in the increased body weight gain [4]. Choline is also a source of methyl groups for carnitine biosynthesis, which, among other things, functions to transport fatty acids from the feed and to mobilize adipose tissue fat from the cytoplasm into the mitochondria to be oxidized into energy to support various biosynthetic processes [5].

The study of the combination of protected KSO treatment with CC is expected to result in a higher increase in meat productivity with higher quality than protected KSO supplementation alone.

This study aimed to examine the influence of protected KSO in combination with CC on the feed utilization, lipid status, and performance of thin-tailed sheep.

## Materials and Methods

### Ethical approval

All the procedures for experimental animal handling were approved by the Welfare and Use Committee of Animal Experiments of the Faculty of Animal and Agricultural Sciences, Diponegoro University, with protocol number 59-01/A-04/KEP-FPP.

### Study period and location

The study was conducted from March to November 2021 at the Feed and Nutrition Laboratory, Faculty of Animal and Agricultural Sciences, Diponegoro University in Semarang, Indonesia.

### Animals and ration

A total of 30 male thin-tailed sheep aged 5–7 months with a body weight of 14.9 ± 1.53 kg were randomly divided into six groups based on the combination of treatments, with each group consisting of five heads as replicates.

The other research materials included KSO (Table-1), protected CC, reagents for KSO protection (KOH, CaCl2, aquadest), field grass, rice bran, and copra meal. The nutritional content of feed ingredients is listed in Table-2, and the composition and nutritional content of standard rations are listed in Table-3.

**Table-1:** Results of KSO analysis.

Variable	Proportion/value
Palmitic acid (C 16:0)	21.97%
Stearic acid (C 18:0)	2.16%
Oleic acid (C 18:1)	23.97%
Linoleic acid (C 18:2)	45.37%
Linolenic acid (C18:3)	4.02%
Saponification number	206.31
Iodin number	11.55

KSO=Kapok seed oil

**Table-2:** Nutrient composition of feedstuff as ration component.

Feedstuff	CP (%)	CF (%)	EE (%)	TDN
Field grass	10.58	35.28	6.71	48.92
Rice bran	13.20	10.29	11.01	81.00
Copra meal	24.67	12.27	9.11	85.07

CP=Crude protein, CF=Crude fiber, EE=Ether extract, TDN=Total digestible nutrients

**Table-3:** Nutrient composition of the standard ration.

Ration component	DM (%)	CP (%)	TDN (%)
Field grass	40	4.23	19.57
Rice bran	31.44	4.15	25.47
Copra meal	28,56	7.05	24.29
Total	100	15.43	69.33

DM=Dry matter, CP=Crude protein, TDN=Total digestible nutrients

### Experimental design and sampling

Treatment factor I was KSO supplementation, consisting of two levels, namely, K_0_ (without supplementation) and K1 (protected KSO supplementation) with supplementation levels of 10% dry matter (DM) rations and 75% protection level, based on the results of research by Widiyanto *et al*. [6]. Treatment factor II was CC supplementation, consisting of three levels, namely, C0 (0%), C_1_ (1.5%), and K_2_ (0.3%), based on the DM of feed. The measured variables included feed consumption, *in vivo* DM digestibility (*Ivo* DMD), *in vivo* organic matter digestibility (*Ivo* OMD), and ration nitrogen retention [7]. The carcass percentage was measured according to Mohammed *et al*. [8]. Triglycerides, low-density lipoprotein (LDL), high-density lipoprotein (HDL), and total cholesterol in blood plasma were determined according to Temple *et al*. [9]. Meat lipid status was analyzed by the extraction method according to Segura *et al*. [10]. The meat samples were dried and homogenized to obtain a uniform powder. The meat samples were extracted in a centrifuge tube after adding dichloromethane. Furthermore, intramuscular fat (IMF) levels were detected by a spectrophotometric method.

The experimental design used was analysis of variance in a 2 × 3 factorial treatment pattern [11]. The collected data were processed by the Costat statistical program.

## Results and Discussion

### Dry matter consumption

Choline chloride supplementation at the level of 3% tended to increase DM consumption (Table-4). The increase in DM consumption was more effective with the combination of protected KSO supplementation. The highest DM consumption (p < 0.05) was achieved in the combination of protected KSO supplementation with a 3% CC level. Protected CC is resistant to rumen microbial degradation so that it is absorbed in the postrumen gastrointestinal tract. Increased absorption of choline increases the synthesis of methionine through the transmethylation process [12]. The increase in amino acid synthesis improves the pattern of amino acids in blood plasma, which can increase appetite so that DM consumption increases [13].

**Table-4:** Dry matter consumption, *Ivo* DMD, *Ivo* OMD, N retention, and DBWG.

Treatment	DMC (g)	*Ivo* DMD (%)	*Ivo* OMD (%)	N retention (g)	DBWG (g)
K_0_C_0_	385.45 ± 5.37^c^	68.33 ± 1.65^bc^	73.08 ± 0.83^b^	6.11 ± 0.24^b^	43.97 ± 1.89^c^
K_0_C_1_	397.09 ± 9.50^c^	69.31 ± 0.43^ab^	75.47 ± 1.04^a^	6.53 ± 0.86^b^	45.97 ± 4.93^c^
K_0_C_2_	428.95 ± 9.17^b^	70.57 ± 0.44^a^	75.85 ± 1.51^a^	6.63 ± 0.49^b^	51.88 ± 0.98^b^
K_1_C_0_	383.07 ± 7.19^c^	70.11 ± 1.46^a^	75.32 ± 1.48^a^	6.46 ± 0.54^b^	50.26 ± 1.58^b^
K_1_C_1_	433.37 ± 10.47^b^	67.30 ± 0.92^c^	72.75 ± 0.89^b^	8.21 ± 0.59^a^	56.38 ± 1.59^a^
K_1_C_2_	448.05 ± 18.51^a^	65.49 ± 1.03^d^	70.97 ± 0.99^c^	8.32 ± 0.46^a^	58.37 ± 1.41^a^

K_0_, K_1_=KSO supplementation, 0 and 10%, respectively, C0, 1, 2=Choline chloride supplementation levels, namely, 0, 1.5, and 3% DM basis, respectively, DMC=Dry matter consumption, *Ivo* DMD=*In vivo* dry matter digestibility, *Ivo* OMD=*In vivo* organic matter digestibility, N retention=Nitrogen retention; DBWG=Daily body weight gain. ^a,b,c^Different superscripts in the same column represent significant differences (p < 0.05)

The increase in DM consumption was even higher in the combination of CC treatment with protected KSO, which showed a significant effect (p < 0.05) on the interaction between CC and protected KSO on DM consumption. Protected KSO supplementation increased the supply of linoleic acid as a biomembrane component that has important biological functions. This allows the activation of intracellular enzymes and an increase in nutrient transport into cells and in turn the metabolic rate of tissues [14]. The increase in the metabolic level combined with the improvement in the pattern of the amino acid composition by increasing the synthesis of methionine increases the biosynthetic process that can increase appetite, so that the increase in DM consumption in the combination of CC treatment with protected KSO is higher [15].

### Digestibility of DM and organic matter

Choline chloride supplementation without the protected KSO combination increased (p < 0.05) both *Ivo* DMD and *Ivo* OMD. The highest digestibility of DM and OM was achieved in the 3% CC supplementation treatment group, which was equivalent to the *Ivo* DMD and *Ivo* OMD in the protected KSO treatment group without CC. The combination with CC supplementation treatment resulted in lower *Ivo* DMD and OMD values than those in the groups without protected CC supplementation, and the lowest digestibility values were observed in the combination treatment with KSO supplementation with 3% CC. Choline can increase the proliferation and performance of rumen microbes when it is the dominant concentrate proportion in the diet. This is suspected to be the cause of the increased DMD and OMD of the rations in the experimental sheep group that received CC supplementation [16]. High DM and OMD values were also achieved in the protected KSO supplementation treatment group without CC supplementation, namely, 70.11 and 75.32%, respectively. The unprotected portion of KSO can provide polyunsaturated fatty acids (PUFAs) for rumen microbes, especially linoleic acid. According to Zhang *et al*. [17], the presence of PUFAs in small amounts can have a positive effect on the digestibility of the ration. This can happen because small amounts of PUFAs are needed by rumen microbes for the synthesis of phospholipids as structural components of rumen microbial cell biomembranes. Approximately 17% of the total constituent of these phospholipids is linoleic acid, which is widely contained in KSO.

Choline chloride supplementation (up to 3%) in the treatment group receiving protected KSO supplements resulted in a decrease (p < 0.05) in *Ivo* DMD and OMD, and the lowest values were found with the combination of protected KSO supplementation with 3% CC (65.49 and 70.97%, respectively). This phenomenon is thought to occur due to a high increase in DM consumption in the groups treated with the combination of protected KSO supplementation and 1.5 and 3% CC. Phesatcha *et al*. [18] stated that increasing the DM consumption could accelerate the flow rate of digesta in the gastrointestinal tract, thus decreasing digestibility.

### Nitrogen retention

Protected KSO supplementation with appropriate rations without CC resulted in N retention that was not significantly different from the N retention in the treatment group without supplementation (control), although the energy/protein ratio in the group was higher than that of the control. On the other hand, CC supplementation without protected KSO also did not significantly increase N retention (3.53 and 3.363 g in the treatment groups without KSO with 1.5 and 3% CC supplementation, respectively) with an energy/protein ratio that was not significantly different, namely, 5.53 and 5.63. The significant increase in N retention (p < 0.05) was only seen in the groups that received combined treatment of KSO supplementation with CC, namely, 8.21 and 8.32 in the combined treatment of KSO supplementation with CC 1.5 and 3%, respectively (Table-4). Nitrogen retention between the two treatment groups was not significantly different, presumably because the energy/protein ratio in the protected KSO supplementation treatment group with 3% CC tended to be lower than the protected KSO supplementation treatment group with 1.5% CC.

### Daily body weight gain (DBWG)

Daily body weight gain tended to increase with CC supplementation at the level of 3%. A significant increase in DBWG occurred with the combination of KSO supplementation, and the highest DBWG value was shown in the 3% CC treatment group with the combination of protected KSO supplementation. Methionine is the first limiting amino acid in ruminants [19]. Choline chloride supplementation increases the supply of methionine by methylation of homocysteine through betaine compounds [20]. Increased methionine will increase the balance of amino acid composition in the tissue, which in turn increases the efficiency of tissue protein biosynthesis [21] and has an impact on increasing DBWG. This occurred mainly in livestock that received rations with inadequate methionine content, such as rice bran and copra meal. This was reflected in the increasing tendency of DBWG in the CC treatment group, namely, from 43.97 g in the control group to 45.57 and 51.88 g in the 1.5 and 3% CC groups, respectively (Table-4).

The increase in the metabolic level, which is supported by an increase in the balance of the composition of essential amino acids in the cells, is more effective in increasing the efficiency of protein biosynthesis. This was reflected in the significant increase in DBWG (p < 0.05) in experimental sheep that received the combination treatment of protected KSO with CC, namely, 56.38 and 58.37 g. The significant increase in DBWG was also supported by an increase in the supply of total digestible nutrients through KSO supplementation to meet the needs of sheep that are growing at a body weight of approximately 15 kg [22].

### Blood lipid status

Supplementation with CC at 1.5% tended to reduce blood plasma cholesterol and the decrease was significant at 3% CC supplementation. This phenomenon presumably occurred because the decreased use of acetic acid, the main precursor of endogenous cholesterol, as the source of energy to support increased protein biosynthesis improved as a result of CC supplementation.

The sheep treated without CC supplementation but supplemented with protected KSO had high total cholesterol levels equivalent to the control, which was 108.51 mg/dL. Cholesterol synthesized in the intestine epithelial cells and liver becomes part of HDLs for transporting lipids from the intestine as well as collecting cholesterol from other lipoproteins and extrahepatic tissue to be oxidized [23]. This process appeared in the experiment, as the levels of LDL cholesterol decreased and the HDL cholesterol increased following the increased level of CC supplementation in the group treated without protected KSO supplementation. The LDL levels in the K_0_C_0_, K_0_C_1_, and K_0_C_2_ treatment groups were 47.58, 43.36, and 41.56 mg/dL, respectively; Moreover, the HDL cholesterol levels were 50.15, 46.33, and 51.78 mg/dL, respectively.

The consumption of lipids in the group treated with protected KSO without CC (K_1_C_0_) was higher than that of the K_0_C_0_ group, but the cholesterol level of the blood plasma between the two treatment groups was not significantly different (108.51 mg/dL and 105.21 mg/dL, respectively). The PUFAs from KSO, after being absorbed, are transported in the form of cholesteryl esters and phospholipids in HDL [24]. Furthermore, the increased HDL is in line with the increased absorption of lipids in the intestine [25]. The level of HDL in the groups treated with protected KSO supplementation without CC was higher than that in the control group and the group supplemented with CC without KSO (Table-5).

**Table-5:** Cholesterol levels in blood plasma of the experimental sheep.

Treatment	Total cholesterol (mg/dL)	LDL (mg/dL)	HDL (mg/dL)
K_0_C_0_	105.21 ± 4.36^a^	47.58 ± 3.21^a^	50.15 ± 1.81^cd^
K_0_C_1_	103.06 ± 5.69^ab^	45.36 ± 3.41^ab^	46.33 ± 1.51^e^
K_0_C_2_	95.29 ± 3.73^c^	41.56 ± 5.99^bc^	51.78 ± 1.13^c^
K_1_C_0_	108.63 ± 3.66^a^	40.35 ± 2.78^c^	59.96 ± 0.97^a^
K_1_C_1_	97.49 ± 3.24^bc^	32.97 ± 1.89^d^	55.60 ± 1.44^b^
K_1_C_2_	84.28 ± 4.73^d^	30.72 ± 2.02^d^	49.60 ± 1.58^d^

K=KSO supplementation, C=Choline chloride supplementation, Col=Cholesterol, ^a,b,c,d^Different superscripts in the same column represent significant differences (p <0.05)

A portion of the PUFAs, mostly in the form of linoleic acid as part of the absorbed lipids, is delivered to various tissues to be used as biological membrane components, and the rest is deposited in adipose tissue, in which PUFAs play less of a role as endogenous cholesterol precursors [25]. Polyunsaturated fatty acids can stimulate cholesterol excretion through the intestine and cholesterol oxidation into bile acids. Polyunsaturated fatty acids also function in reducing cholesterol by increasing LDL catabolism and the number of LDL receptors [26].

Supplementation with protected KSO increased the supply of linoleic acid that, among other functions, acted as a structural part of the biological membrane and as a second messenger. This process activates adenyl cyclase enzyme, which catalyzes the conversion of adenosine triphosphate into cyclic adenosine monophosphate [23]. The latter compound activates kinase protein, which in turn increases intracellular metabolism [27].

The decreased levels of total blood cholesterol in the combined KSO supplementation with CC at levels of 1.5 and 3% were 97.59 mg/dL and 84.28 mg/dL, respectively, in the K_1_C_1_ and K_1_C_2_ treatment groups compared to the K_1_C_0_ treatment group, which was 108.51 mg/dL. A decrease (p < 0.05) also occurred in the LDL cholesterol levels in the K_1_C_0_ treatment group from 40.35 mg/dL to 36.17 and 30.72 mg/dL, respectively.

The increased metabolic rate requires HDL functioning to supply cholesterol to be oxidized in extrahepatic tissue as an energy source; in the liver, cholesterol is used to synthesize bile acid for lipid digestion, and its supply increases in line with the protected KSO supplementation [28]. Given this mechanism, an interesting phenomenon was identified in the K_1_C_1_ and K_1_C_2_ treatment groups; the HDL cholesterol levels of the K_1_C_1_ and K_1_C_2_ groups were even lower than that of the K_1_C_0_ group (55.60 mg/dL and 49.60 mg/dL vs. 59.96 mg/dL). This phenomenon occurred because there was an increased use of cholesterol precursors to increase energy supply and steroidogenesis to support increased protein anabolism [29]. This process could be observed in the increased ratio of HDL cholesterol to LDL cholesterol, which was manifested in the combination K_1_C_1_ and K_1_C_2_ treatment groups compared to the ratio in the combination treatment K_1_C_0_ group.

### Meat lipid status

The lipid status of the experimental animals could be assessed by the levels of IMF and cholesterol as well as the relative proportion of meat linolenic acid. Data on these variables are presented in Table-6. The IMF levels in the treatment group without protected KSO supplementation at CC supplementation levels of 0% (K_0_C_0_), 1.5% (K_0_C_1_), and 3% (K_0_C_2_) were 5.95%, 5.78%, and 4.55%, respectively. A tendency toward decreased levels of IMF occurred in the combination of K_0_C_1_ compared to the control; moreover, a decrease (p < 0.05) also occurred in the K_0_C_2_ treatment combination.

**Table-6:** Levels of IMF, cholesterol, and relative proportions of linoleic acid in the musculus longissimus thoracis of the experimental sheep.

Treatment	IMF^1)^ (%)	Cholesterol^2)^ (mg/100 g)	Linoleic acid^3)^ (%)
K_0_C_0_	5.92 ± 0.90^a^	89.37 ± 0.59^a^	4.98 ± 0.08^d^
K_0_C_1_	5.78 ± 0.26^a^	87.63 ± 1.38^ab^	6.41 ± 1.71^d^
K_0_C_2_	4.55 ± 0.36^b^	83.21 ± 6.75^b^	8.09 ± 1.58^c^
K_1_C_0_	5.53 ± 0.51^a^	70.69 ± 3.97^c^	8.4 ± 0.85^c^
K_1_C_1_	3.98 ± 0.22^b^	67.18 ± 2.79^c^	15.87 ± 1.32^b^
K_1_C_2_	2.93 ± 0.56^c^	60.95 ± 2.02^d^	18.08 ± 1.19^a^

K=KSO supplementation, C=CC supplementation,

1) =Percentage of fresh meat weight,

2) =mg cholesterol per 100 g of fresh meat,

3) =Percentage of linoleic acid from total fatty acids of meat, ^a,b,c,d^Different superscripts in the same column represent significant differences (p < 0.05), IMF=Intramuscular fat

Intramuscular fat is an esterification product that requires *de novo* fatty acid synthesis and the absorption of long-chain fatty acids from feed, as well as alpha-glycerol phosphate generated as a reduction compound formed during glycolysis from glucose, in the form of dihydroxyacetone phosphate catalyzed by the enzyme glycerol phosphate dehydrogenase [30]. Glucose derived from propionic acid gluconeogenesis, mainly from ruminal digestible carbohydrate fermentation, is also a source of hydrogenated nicotinamide adenine dinucleotide phosphate, which functions as a reducing agent in the biosynthesis of *de novo* fatty acids as well as an energy source [31]. The main precursor of *de novo* fatty acids is acetic acid, which is a product of ruminal fermentation from both digestible carbohydrates and, mainly, fiber [32]. The increased availability of methionine as a limiting amino acid, in this case through CC supplementation, increases the use of propionic acid and/or glucose to support the biosynthesis of tissue proteins and decreases their use for the biosynthesis of *de novo* fatty acids and their esterification, thereby reducing IMF deposition [30].

Lipid consumption in the group treated with protected KSO supplementation was higher than that in the group treated without protected KSO supplementation. An interesting phenomenon occurred, as the IMF level of the K_1_C_0_ treatment group was not significantly different from the IMF level of the K_0_C_0_ treatment group (5.53% vs. 5.95%). This result occurred because of the inhibition of *de novo* fatty acid biosynthesis by PUFAs so that their deposition as part of IMF was low. The low biosynthesis of *de novo* fatty acids can occur due to inhibition of RNA transcription that regulates the synthesis of the acetyl-CoA carboxylase enzyme by PUFAs [31]. Acetyl-CoA carboxylase is an enzyme that catalyzes the carboxylation of acetyl-CoA as an initial step in the biosynthesis of *de novo* fatty acids [33]. The combination of protected KSO supplementation and CC increased the use of volatile fatty acids and glucose as carbon frameworks and energy in protein biosynthesis to foster a decrease in *de novo* fatty acid biosynthesis. The mechanism appeared to involve the IMF levels in the K_1_C_1_ and K_1_C_2_ treatment groups, which were lower than those of the other treatment groups (p < 0.05).

Choline chloride supplementation alone did not significantly affect the cholesterol levels of the meat, although there was a tendency toward a decrease in the cholesterol levels. The tendency of the meat cholesterol level to decrease was presumed to be due to an increase in lipid oxidation as an energy source to support protein biosynthesis, which could be measured by an increase in N retention and DBWG (Table-4).

A significant decrease in the meat cholesterol levels appeared in the group treated with protected KSO supplementation, and the decrease was even greater in the groups that were treated in combination with CC (p < 0.05). The cholesterol levels of the meat in the groups treated with protected KSO in combination with CC at the level of 0%, 1.5%, and 3% were 70.69, 67.18, and 58.71 mg/100 g, respectively (Table-6).

Supplementation with polyunsaturated fatty acids from KSO increased their availability as one of the precursors of the constituent compounds in biomembranes and the second messengers that maintained membrane integrity and activation of intracellular metabolism [34]. The PUFA potency in intracellular metabolism, combined with CC supplementation, which serves to increase one of the essential amino acid precursors, will enhance protein anabolism, which requires support in the form of a higher energy supply [4]. The need for a higher energy supply led to the oxidation of compounds, including carbohydrates and lipids, resulting in a decrease in meat cholesterol levels [35]. This process underlined the fact that a lower level of meat cholesterol was found in the group treated with CC supplementation at the 3% level in combination with 10% protected KSO from digestible DM at the 75% protected level.

According to Toral *et al*. [36], ruminants selectively incorporate PUFAs, mainly C18:2, n-6 or linoleic acid, into phospholipids, not into triglycerides. Incorporation into phospholipids is a conservation mechanism of PUFAs because phospholipid metabolism is slower than that of triglycerides. Most of the phospholipids are then deposited as part of the structural membrane of muscle tissue, whereas most IMF is deposited in the intramuscular adipose tissue.

In this experiment, in the treatment group that was not supplemented with protected KSO, the decreased proportion of the IMF was in line with the increased level of CC supplementation, indicating that an increase in the proportion of meat muscle tissue took place. Therefore, the increased proportion of linoleic acid (p < 0.05) was in line with the increased level of CC, as most of the available linoleic acid became a structural part of the membrane cell of the meat muscle tissue. This is shown in Table-6, where the relative proportion of linoleic acid in the treatment group without protected KSO supplementation that supplemented with CC at levels of 0%, 1.5%, and 3% was 4.98%, 6.41%, and 8.09%, respectively.

The combined effect of CC supplementation with protected KSO was even greater when increasing the proportion of muscle tissue as a result of the increased level of metabolism supported by essential amino acid intake, as shown by the increase (p < 0.05) in N retention and DBWG (Table-4). This phenomenon resulted in an increase in the relative proportion of linoleic acid (p < 0.05), which was in line with the increased level of the CC in the protected KSO supplementation treatment group, which was 8.4%, 15.87%, and 18.08% at CC levels of 0, 1.5, and 3%, respectively (Table-6).

## Conclusion

Protected KSO supplementation at the 10% level combined with CC at the 3% level increased the performance of male thin-tailed sheep. The performance improvement was in the form of increased feed consumption and DBWG as well as meat quality, which was reflected in the decreased levels of cholesterol and IMF and increased relative proportions of linoleic acid. This treatment also improved blood lipid status, that is, it decreased total cholesterol and LDL cholesterol levels and increased HDL cholesterol levels.

## Authors’ Contributions

WW: Designed the study. WW, BWHEP, and MM: Drafted the manuscript, participated in conducting the experiment, performed the *in vivo* experiment and biochemical investigation, collected the samples, and processed and analyzed the data. All authors have read, reviewed, and approved the final manuscript.
